# Pharmacological suppression of the WNT signaling pathway attenuates age-dependent expression of the phenotype in a mouse model of arrhythmogenic cardiomyopathy

**DOI:** 10.20517/jca.2021.04

**Published:** 2021-06-06

**Authors:** Sirisha M. Cheedipudi, Siyang Fan, Leila Rouhi, Ali J. Marian

**Affiliations:** Center for Cardiovascular Genetics, Institute of Molecular Medicine and Department of Medicine, University of Texas Health Sciences Center at Houston, Houston, TX 77030, USA.

**Keywords:** Cardiomyopathy, WNT signaling, fibrosis, apoptosis, heart failure

## Abstract

**Introduction:**

Arrhythmogenic cardiomyopathy (ACM) is a genetic disease of the myocardium, characterized by cardiac arrhythmias, dysfunction, and sudden cardiac death. The pathological hallmark of ACM is fibro-adipocytes replacing cardiac myocytes. The canonical WNT pathway is implicated in the pathogenesis of ACM.

**Aim:**

The study aimed to determine the effects of the suppression of the WNT pathway on cardiac phenotype in a mouse model of ACM.

**Methods and Results:**

One copy of the *Dsp* gene, a known cause of ACM in humans, was deleted specifically in cardiac myocytes (*Myh6-Cre*-*Dsp*^W/F^). Three-month-old wild type and *Myh6-Cre*-*Dsp*^W/F^ mice, without a discernible phenotype, were randomized to either untreated or daily administration of a vehicle (placebo), or WNT974, the latter an established inhibitor of the WNT pathway, for three months. The *Myh6-Cre*-*Dsp*^W/F^ mice in the untreated or placebo-treated groups exhibited cardiac dilatation and dysfunction, increased myocardial fibrosis, and apoptosis upon completion of the study, which was verified by complementary methods. Daily administration of WNT974 prevented and/or attenuated evolving cardiac dilatation and dysfunction, normalized myocardial fibrosis, and reduced apoptosis, compared to the untreated or placebo-treated groups. However, administration of WNT974 increased the number of adipocytes only in the *Myh6-Cre*-*Dsp*^W/F^ hearts. There were no differences in the incidence of cardiac arrhythmias and survival rates.

**Conclusion:**

Suppression of the WNT pathway imparts salutary phenotypic effects by preventing or attenuating age-dependent expression of cardiac dilatation and dysfunction, myocardial fibrosis, and apoptosis in a mouse model of ACM. The findings set the stage for large-scale studies and studies in larger animal models to test the beneficial effects of the suppression of the WNT pathway in ACM.

**One sentence summary:**

Suppression of the WNT signaling pathway has beneficial effects on cardiac dysfunction, myocardial apoptosis, and fibrosis in a mouse model of arrhythmogenic cardiomyopathy.

## INTRODUCTION

Arrhythmogenic cardiomyopathy (ACM) is a primary myocardial disease that manifests with cardiac arrhythmias, sudden cardiac death (SCD), and progressive heart failure^[1]^. Cardiac arrhythmias are often the first manifestation of ACM, typically occurring between the second to fourth decades of life, and, on occasion, manifesting as SCD^[2]^. Heart failure usually manifests later and is progressive, often necessitating heart transplantation^[2]^. Cardiac arrhythmias also occur late in ACM course in conjunction with advanced heart failure, as in other forms of heart failure^[2]^.

ACM encompasses a heterogenous group of myocardial diseases, whose distinction from dilated cardiomyopathy (DCM), is the predominance of ventricular arrhythmias, as opposed to cardiac dysfunction and heart failure, which are the main phenotypic features of DCM. The classic ACM form is called arrhythmogenic right ventricular cardiomyopathy (ARVC) because it predominantly involves the right ventricle. ARVC typically manifests with ventricular arrhythmias originating from the right ventricular outflow tract and is pathologically characterized by fibro-fatty infiltration of the myocardium. The latter exhibits a predilection toward the involvement of the right ventricle, albeit, in the advanced stages, the left ventricle is also involved^[3,4]^. A subgroup of ACM predominantly and on occasion exclusively involves the left ventricle and is referred to as arrhythmogenic left ventricular cardiomyopathy (ALCM) or left-dominant ACM^[5]^.

The molecular genetic basis of ACM has been partially elucidated upon identification of pathogenic variants (PVs), commonly referred to as mutations, in several genes encoding the intercalated disc, particularly desmosome proteins. *PKP2*, *DSP*, *DSG2*, *DSC2*, and *JUP*, encoding plakophilin 2, desmoplakin, desmoglein 2, desmocolin 2, and junction protein plakoglobin, respectively, are the most common causal genes in ACM^[6–10]^. A notable feature of ACM caused by the PVs in the *DSP* gene is a predilection toward ALCM with pronounced myocardial fibrosis, often resembling DCM^[11]^. PVs in genes encoding other cell adhesion proteins, such as cadherin 2 (CDH2) and αT-catenin (CTNNA3), as well as an actin-binding protein filamin C (FLNC), also have been identified as causes of ACM^[12–14]^. Moreover, PVs in the *PLN* gene, which encodes the caclium handling protein phospholamban, are implicated in ACM, particularly, ALCM^[15]^. Similarly, PVs in *RBM20*, which encodes a splicing protein that targets sarcomere genes, are also implicated in ACM^[16]^. Finally, PVs in genes encoding nuclear envelope proteins lamin A/C (LMNA) and transmembrane protein 43 (TMEM43) are important causes of ACM. Collectively, the discoveries point to the genetic and phenotypic heterogeneity of the ACM^[17,18]^.

The molecular pathogenesis of ACM has remained mostly elusive. Given the key role of the intercalated disc proteins in maintaining the mechanical integrity of the myocardium through cell-cell adhesion, pathways involved in mechanosensing and mechanotransduction, such as the integrins, the canonical WNT (cWNT), and the Hippo pathways, have emerged as the prime contributors to the pathogenesis of ACM^[19–24]^. Experimental data suggest the suppression of the cWNT pathway in the myocardial tissue in ACM models and has reported inconsistent findings on the effects of activation of the cWNT pathway on the cardiac phenotypes^[21,25–29]^. Thus, the purpose of the study was to determine the phenotypic effects of the suppression of the WNT pathway in a mouse model of ACM caused by *Dsp* haploinsufficiency. The findings are expected to provide guidance for the translational applications of interventions designed to target the WNT pathway in ACM.

## METHODS

### Regulatory approval

The animal experiments were conducted according to the NIH Guide for the Care and Use of Laboratory Animals and were approved by the Institutional Care and Use Committee (Protocol # AWC-18–0048).

### *Myh6-Cre:DspW/F* mice

Cardiac myocyte-specific *Dsp* haploinsufficient (*Myh6-Cre:Dsp*^W/F^) mice were generated by crossing the *Myh6-Cre* and *Dsp*^F/F^ mice, as published^[21,30]^. Littermate wild-type (WT) mice were used as controls in the experiments.

### Genotyping

Genotyping was performed as described^[21,31]^. In brief, genomic DNA was extracted from the tail tissues upon overnight digestion in a lysis buffer containing proteinase K at 55 °C (Qiagen Cat# 158267). After the precipitation of the protein fraction, the supernatant was transferred to a fresh tube containing isopropanol. DNA was pelleted upon centrifugation at 13,000 rpm for 10 min at room temperature. The pelleted DNA was washed once with 70% ethanol and was re-precipitated by centrifugation at 13,000 rpm for 5 min at room temperature and then air-dried. Finally, the DNA was dissolved in water, and a PCR reaction was performed using 100 ng of DNA. The oligonucleotide primers used for genotyping are listed in Supplementary Table 1.

### Suppression of the WNT signaling pathway

To determine the effects of the suppression of the WNT signaling pathway on cardiac phenotype, a randomized placebo-controlled study was performed whereby the WT or *Myh6-Cre:Dsp*^W/F^ mice were treated either with a placebo or the WNT974 (formerly known as LGK-974). The latter inhibits porcupine, a key post-translational enzyme necessary for signaling through the WNT pathway^[32]^. Porcupine is a membrane-bound acetyltransferase that palmitoylates the WNT molecules and enables transmembrane transport and extracellular secretion of the WNT molecules^[32]^.

*WT* and *Myh6-Cre:Dsp*^W/F^ mice were randomized to either no therapy or daily subcutaneous injection of a placebo (8% DMSO in sesame oil, as the vehicle) or WNT974, which was dissolved in sesame oil containing 8% DMSO. The randomization was started in 3 months old mice, as the *Myh6-Cre:Dsp*^W/F^ do not show a discernible phenotype at this age, except for a modest increase in myocardial apoptosis^[30]^. The intervention was continued for 3 months, as upon completion of the study, the 6 months *Myh6-Cre:Dsp*^W/F^ mice are expected to exhibit cardiac dysfunction and fibrosis^[21,31]^. The dose of WNT974 was selected based on the published data^[32,33]^.

### Survival curve

Kaplan Meier survival rates were determined in all 6 study groups, and plotted using GraphPad Prism 8 software (https://www.graphpad.com/).

### Gross morphology

Body weight and heart weight/body weight ratio were measured upon completion of the study and the mean values were compared among the groups.

### Echocardiography

Echocardiography was performed using a Vevo 1100 ultrasound imaging system (FUJIFILM VisualSonics Inc., Toronto, ON, Canada), as published^[30,34]^. In brief, mice were lightly anesthetized using 1% isoflurane to maintain a heart rate of around 500 bpm. B-mode guided M mode images were obtained. The leading-edge method was used to measure anterior wall thickness (AWT), posterior wall thickness (PWT), left ventricular end systolic diameter (LVESD), and left ventricular end diastolic diameter (LVEDD). Left ventricular fractional shortening (LVFS) and left ventricular mass were calculated from the above measurements and the latter indexed to body weight.

### Electrocardiography

Cardiac rhythm was monitored for about an hour in each mouse using surface electrodes while the mouse was kept under light anesthesia, as published^[35,36]^. The rhythm was recorded using a Power Lab 4/30 data acquisition system and data were analyzed using Lab Chart7 software (ADInstruments, Colorado Springs, CO, USA).

### Picrosirius red staining

The heart was removed and fixed in 10% neutral buffered formalin overnight at 4 °C. The heart tissue was dehydrated in a gradient of ethanol concentrations of 70%, 80%, 95%, and 100%, followed by incubation in xylene for 15 min and embedding in paraffin blocks at 60 °C for 1 h. Thin (5 μm) myocardial sections were cut using a microtome (Leica), placed onto a Super frost microscopic slides (VWR, Cat# 48311–703), and left at 37 °C overnight to dry.

To detect and quantify myocardial fibrosis, thin myocardial sections were deparaffinized with xylene for 15 min twice at room temperature and were rehydrated in a series of ethanol concentrations of 100%, 95%, 80%, 70%, and 50% for 2 min each at room temperature. The sections were stained with picrosirius red according to the manufacturer’s protocol (Sigma Aldrich, Cat# P6744–1GA), mounted in a xylene based mounting medium, and were imaged using an Olympus microscope equipped with a digital camera (Olympus BX40). Collagen volume fraction (CVF) was calculated in 15–20 high microscopic fields (×20) per section, 6 sections per heart, and in a minimum of 5 mice per group using Image J software, as published^[30,36]^.

### Immunofluorescence

The heart was excised, embedded in an OCT-containing compound, placed immediately in 2-methyl butane, and was flash-frozen in liquid nitrogen. Thin myocardial sections (5 μm) were cut using a cryostat and fixed in 4% formaldehyde at room temperature for 10 min. Myocardial sections were washed at room temperature twice with PBS (1×) for 10 min each. Following washing, the tissue sections were permeabilized using PBS and 0.5% Triton-X-100 for 30 min at room temperature. Sections were then incubated with a blocking buffer containing PBS, 0.5% Triton-X-100, 10% goat serum, and 1% BSA for 1 h at room temperature, followed by incubation with the primary antibody of interest.

To detect adipocytes in the myocardium, thin myocardial sections were subjected to antigen retrieval upon treating the sections with sodium citrate (10 mM, pH 6.0) and boiling for 20 min at 95 °C. Following antigen retrieval, the sections were incubated with an antibody against Perilipin 1 (PLIN1), a marker of lipid-containing adipocytes (Cell Signaling Technology Cat# 9349), at 1:100 dilution overnight at 4 °C, as published^[36]^. Sections were then washed for 3 times for 15 min each with PBS and 0.5% Triton-X-100 and were incubated with the secondary antibody conjugated to Alexa 594 for 1 h at room temperature. After three washes with PBS and 0.5% Triton-X-100 for 15 min each, nuclei were counter stained with 4’, 6 Diamidino-2-phenylindole dihydrochlorides (DAPI, Sigma-Aldrich St Louis, MO; Cat# D8417) at 1 μg/mL concentration for 10 min at room temperature and mounted using fluorescence mounting media (DAKO, Cat# S3023). The number of cells expressing PLIN1 was counted in each myocardial section, in 6 sections per heart, and in a minimum of 5 mice per group.

To detect expression of TCF7L2, the main transcriptional regulator of the cWNT pathway, immunostaining of thin myocardial sections was performed as described above, except for using an anti TCF7L2 antibody (Cell Signaling Technology Cat# 2569). Immunofluorescence images were acquired using Zeiss Axioplan fluorescence microscope from 10–15 high magnification fields (×40), five sections per heart, and a minimum of 5 hearts per genotype. Approximately 7000 to 15,000 DAPI positive nuclei were counted per mouse using Image J software, and the percentage of nuclei stained positive for the expression of TCF7L2 protein was calculated.

### Wheat germ agglutinin staining

To determine myocyte cross-sectional area, thin myocardial sections were stained with wheat germ agglutinin (WGA) as published^[30,36]^. Briefly, 5 μm thin paraffin sections were incubated with WGA (1 μg/mL) conjugated to Texas red (Thermo Fisher Scientific, Cat# W21405). To identify cardiac myocytes, sections were co-stained with an antibody against pericentriolar material protein 1 (PCM1), which specifically marks myocytes in the heart, at 1:1000 dilution in a blocking buffer overnight at 4 °C (Sigma Cat# HPA023370)^[30,36–38]^. Sections were then stained with DAPI, mounted using a fluorescent mounting media, and imaged, as described above. WGA-stained images were analyzed using Image J software (https://imagej.net). Total pixels obtained for WGA staining were subtracted from the total pixel count per field. The residual pixel count was divided by an average number of cardiac myocytes identified by PCM1 staining in each field. An average of 10 to 15 fields per section, 5–6 sections per heart, representing around 15,000 to 20,000 nuclei per heart. About 4–7 mice per genotype were used to calculate the mean myocyte cross-sectional area.

### TUNEL assay

Apoptosis in the myocardium was detected using the terminal deoxynucleotidyl transferase dUTP nick end labelling (TUNEL) assay by *in situ* cell death detection Fluorescein kit (Roche Cat# 11684795910), as published^[30,36]^. In brief, thin myocardial sections were deparaffinized in xylene baths, rehydrated in a gradient of ethanol concentration, as described above, were treated with proteinase K (20 μg/mL in 10 mM Tris-Cl pH 7.5 containing 1 mg/mL BSA) for 20 min at room temperature. The sections were then incubated with the TUNEL reaction mix for 1 h at room temperature, and the nuclei were counterstained with DAPI and imaged, as described above. The percentage of the nuclei stained positive for TUNEL was calculated in 15 high magnification fields (×40) per section, in 5–6 sections per heart, and in 4 to 7 mice per group. Approximately 15,000 to 24,000 nuclei were counted per mouse heart, and the mean values were compared among the groups.

### Reverse transcription-polymerase chain reaction

Total RNA was isolated from whole heart tissue using a miRNeasy Mini Kit (Qiagen, Cat# 217006) and treated with DNAse I to remove the genomic DNA (Qiagen, Cat# 79254) as per the manufacturer’s instructions. An aliquot of 1 μg of total RNA was used to perform reverse transcription using a high-capacity cDNA synthesis kit with random primers (Applied Biosystems Cat# 4368814). Transcript levels of the selected genes were determined by reverse transcription-polymerase chain reaction (RT-PCR) using SYBR green or TaqMan assays and were normalized to *Gapdh* mRNA levels, as described^[34]^. Changes in the transcript levels were determined as fold changes using the ΔCT method and presented as relative levels compared to the values in the WT untreated mouse group. All RT-PCR experiments were performed in duplicates and in 5–7 mice per each group. The primers used in the RT-PCR reactions are listed in the Supplementary Table 1.

### Statistical methods

Shapiro-Wilk normality test was used to assess Gaussian distribution of the data. Data that followed a Gaussian distribution (normally distributed) were compared between two groups using the *t*-test and among multiple groups by one-way ANOVA followed by Bonferroni pairwise comparisons. Data that departed from the Gaussian distribution were analyzed by the Kruskal-Wallis test. The categorical data were analyzed by the Fisher exact or the Chi-Square test. Genotype-by-treatment interaction analyses were performed by two-way ANOVA. Kaplan-Meier survival plots were constructed and compared using the Log-rank test. Statistical analyses were performed using Graph pad Prism 8 or STAT IC, 15.1.

## RESULTS

### Suppression of the WNT pathway

**Figure 1.**
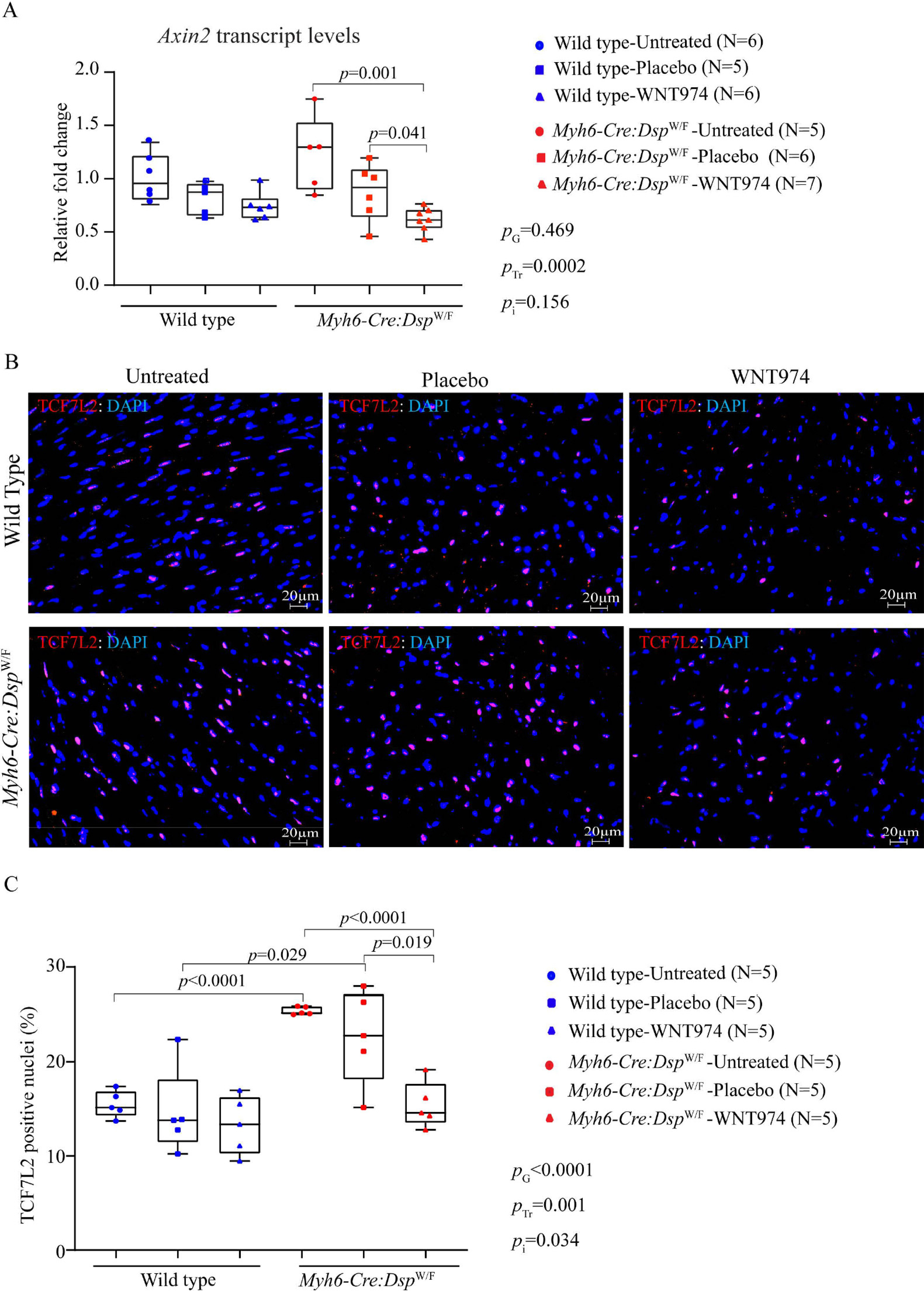
Suppression of the canonical WNT in the wild type and *Myh6-Cre:Dsp*^W/F^ (cardiac myocyte-specific heterozygous deletion of the *Dsp* gene) mice. (A) Transcript levels of *Axin2*, a bona fide target of the cWNT pathway in the wild type and *Myh6-Cre:Dsp*^W/F^ mice treated with placebo and WNT974 along with untreated controls, as determined by RT-PCR. Relative fold change in the transcript levels was determined by comparing all the groups to the wild type untreated group. (*N* = 5–7 per group). (B) Representative immunofluorescence images depicting expression and nuclear localization of TCF7L2 protein, a co-transcriptional regulator of the canonical WNT pathway, in the myocardial sections in the experimental groups. (C) Quantitative data showing the percentage of cells expressing TCF7L2 in the myocardial sections in the experimental groups. The *P* values were determined by the student *t*-test between two groups and genotype-by-treatment interactions were determined by 2-way ANOVA. The significant *P* values (< 0.05) between the groups are presented. *P*_G_: *P* value for the effect of the genotype; *P*_Tr_: *P* value for the effect of treatment; *P*_i_: *P* value for treatment-by-genotype interaction.

To determine whether daily administration of WNT974 suppressed the WNT pathway, myocardial transcript levels of *Axin2*, a well-established bona fide target of the cWNT pathway, were quantified by RT-PCR. Systemic administration of WNT974 reduced *Axin2* transcript levels by 26% ± 13% in the WT and 61% ± 11% in the *Myh6-Cre:Dsp*^W/F^ mouse hearts, respectively, as compared to the corresponding levels in the untreated groups [Figure 1A]. Treatment-by-genotype interaction analysis showed the genotype-independent effect of treatment with WNT974 in reducing *Axin2* transcript levels, albeit the reduction in the *Myh6-Cre:Dsp*^W/F^ mouse hearts was more pronounced [Figure 1A].

To further assess the effectiveness of the WNT974 in suppressing the WNT pathway, nuclear localization of TCF7L2, the main transcriptional regulator of the cWNT pathway, was analyzed upon immunofluorescence staining of thin myocardial sections. Treatment with WNT974 markedly reduced nuclear localization of TCF7L2, as indicated by the reduced number of nuclei stained positive for the expression of *TCF7L2* in the *Myh6-Cre:Dsp*^W/F^ mouse hearts [Figure 1B and C]. The effect of treatment with WNT974 was remarkable in the *Myh6-Cre:Dsp*^W/F^ mouse hearts [Figure 1C]. Interaction analysis showed significant effects of the genotypes as well as the treatment with WNT974 on the number of cells showing expression of the *TCF7L2* transcription factor [Figure 1C].

### Survival and gross morphology

There were no significant differences in the survival rates during the 3 months study period among the experimental groups [Supplementary Figure 1A]. Likewise, the body weight and the heart weight/body weight ratios upon completion of the study at 6 months of age were similar among the groups [Supplementary Figure 1B and C].

### Cardiac size and function

To determine the effects of the suppression of the WNT pathway on cardiac size and function, echocardiography was performed before the start of randomization and upon completion of the study. Prior to the initiation of therapy, there were no significant differences in echocardiographic indices of cardiac size and function, including LVEDD, LVESD, and LVFS among the experimental groups [Supplementary Table 2]. Upon completion of the intervention, i.e., at 6 months of age, the *Myh6-Cre:Dsp*^W/F^ mice in the untreated or the placebo group had developed cardiac dilatation and dysfunction, as compared to the corresponding *WT* mice, as evidenced by increased LVEDD, LVESD, and reduced FS, which are consistent with the published data [Table 1]^[21,30]^. Treatment with WNT974 had no significant effect on indices of cardiac dilatation and dysfunction in the *WT* mice [Table 1]. In contrast, daily administration of WNT974 attenuated cardiac dilatation and dysfunction in the *Myh6-Cre:Dsp*^W/F^ mice [Table 1]. Accordingly, the LVFS was improved from 32.4% ± 8.6% in the untreated to 40.7% ± 5.5% in WNT974 treated *Myh6-Cre:Dsp*^W/F^ mice, which was similar to that in the *WT* mice [Table 1]. Treatment-by-genotype interaction analysis showed significant effects of the genotype on indices of LV size and LVFS as well as the beneficial effects of WNT974 treatment on LVFS [Table 1].

**Figure 2.**
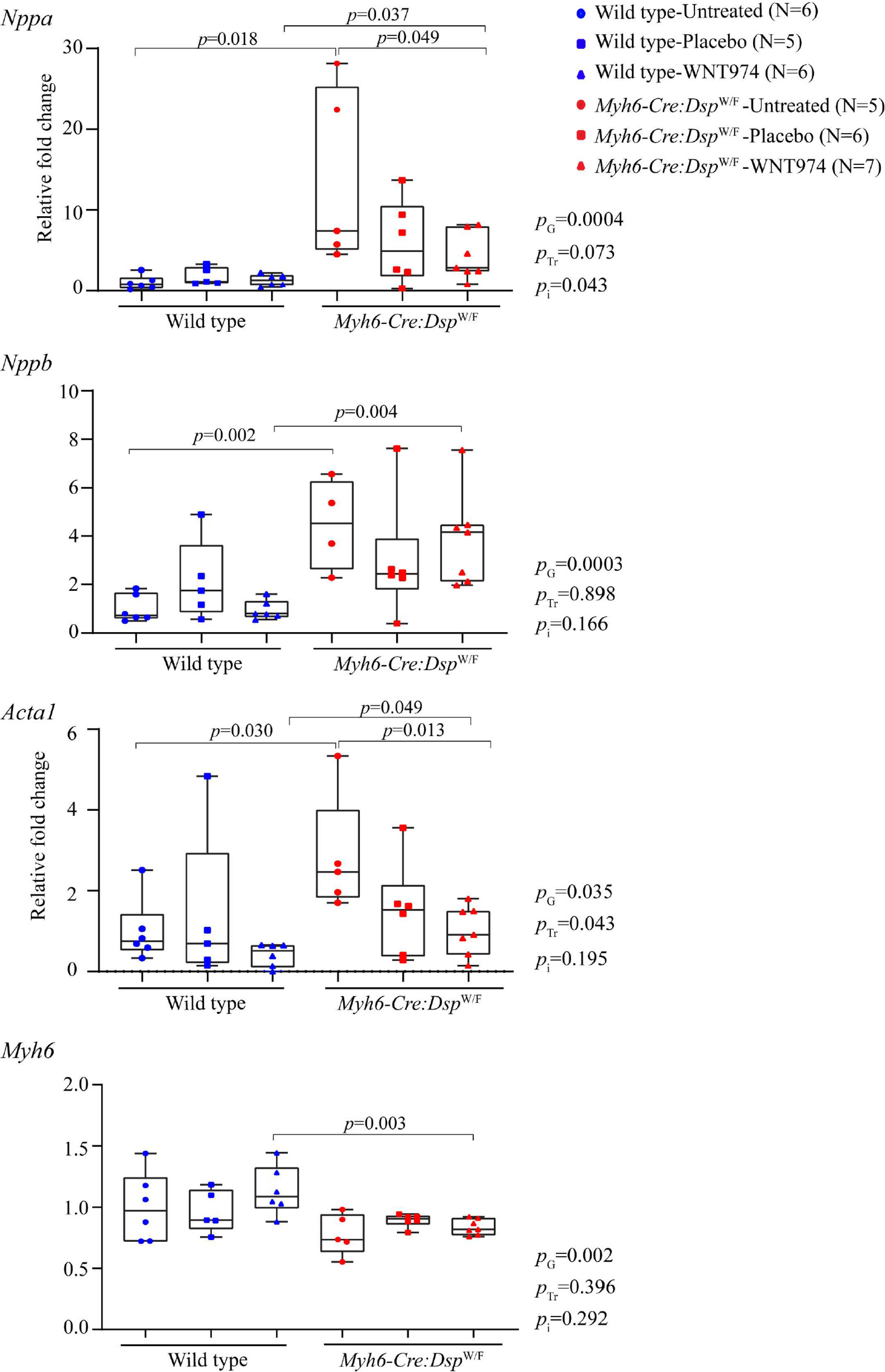
Partial rescue of transcript levels of markers of cardiac dysfunction in the *Myh6-Cre:Dsp*^W/F^ mice upon suppression of the cWNT. Quantitative RT-PCR data depicting the transcript levels of markers of cardiac dysfunction *Nppa* (A type natriuretic peptide), *Nppb* (B-type natriuretic peptide), *Acta1* (actin α, skeletal), and *Myh6 Myh6* (myosin heavy chain 6) in the wild type and *Myh6-Cre:Dsp*^W/F^ mice treated with WNT974 as compared to the untreated and placebo treated mice. *P*_G_: *P* value for the effect of the genotype; *P*_Tr_: *P* value for the effect of treatment; *P*_i_: *P* value for treatment-by-genotype interaction.

Concordant with cardiac dysfunction in the *Myh6-Cre:Dsp*^W/F^ mice and improvement of function upon suppression of the WNT pathway, transcript levels of markers of cardiac dysfunction and failure, including *Nppa*, *Nppb* and *Acta1* were significantly upregulated, whereas transcript levels of *Myh6* were reduced in the *Myh6-Cre:Dsp*^W/F^ mouse hearts as compared to the WT [Figure 2]. Treatment with WNT974 reversed and normalized elevated levels of *Nppa* and *Acta1* transcripts in the *Myh6-Cre:Dsp*^W/F^ mouse hearts, whereas levels of *Nppb* and *Myh6* transcripts were unchanged [Figure 2]. Likewise, genotype-by-treatment interactions showed marked effects of the genotype on the transcript levels of the selected markers of cardiac hypertrophy and dysfunction.

### Cardiac arrhythmias

Only occasional ventricular ectopic beats, sinus brady-arrhythmias, and episodes of sinus pause were detected in *Myh6-Cre:Dsp*^W/F^ mice. There were no significant cardiac arrhythmias prior to the onset of therapy and after 3 months of therapy in mice in any of the experimental groups.

### Myocardial fibrosis

**Figure 3.**
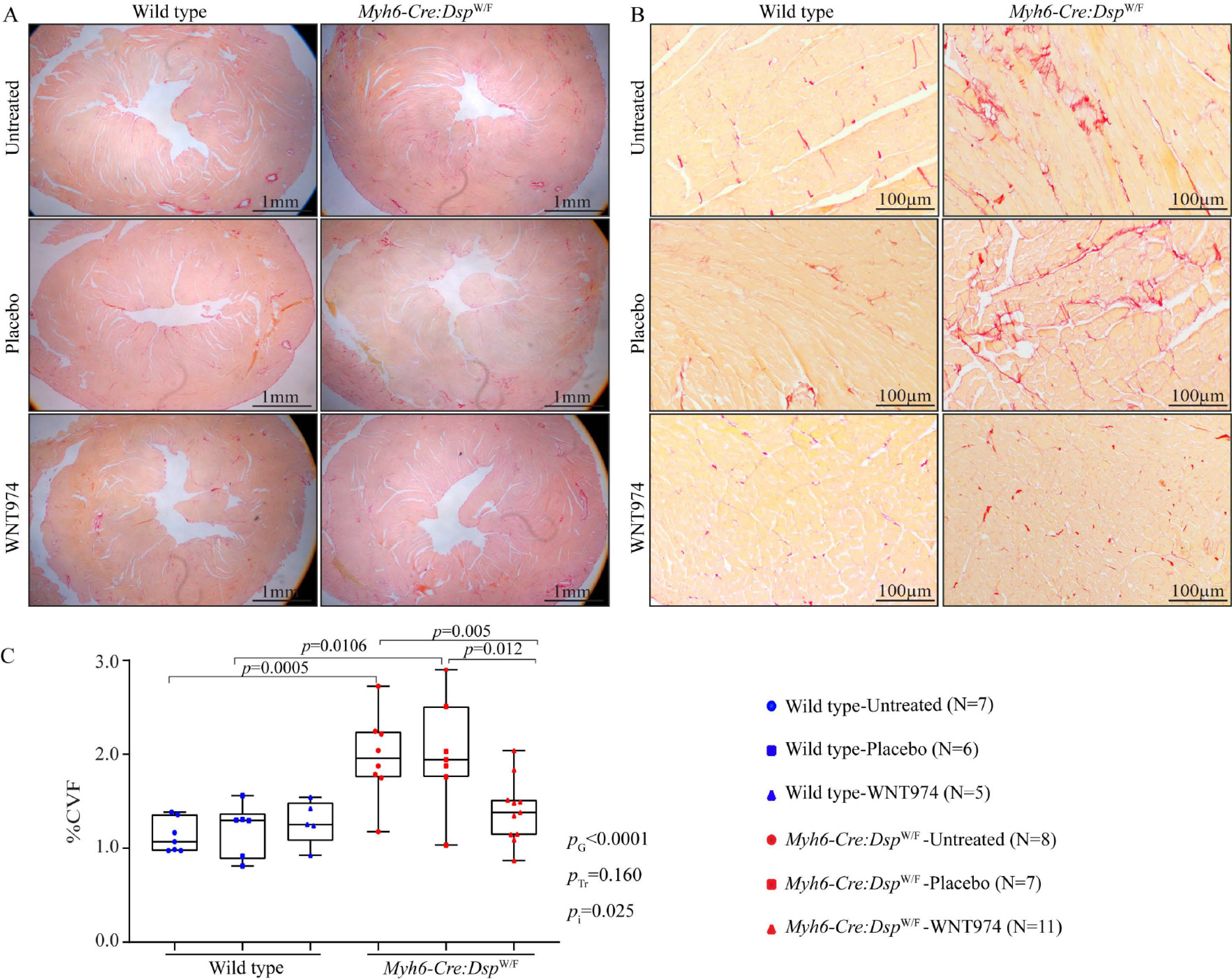
Effects of suppression of the cWNT pathway on myocardial fibrosis. (A) Low magnification images depicting picrosirius red stained thin myocardial sections in the wild type and *Myh6-Cre:Dsp*^W/F^ mice, either untreated, and treated with a placebo or WNT974. (B) High magnification images depicting thin myocardial section stained with picrosirius red in the experimental groups. (C) Bar graphs showing collagen volume fraction (CVF), as determined by the morphometric analysis of at least 6 high magnification field per section, 6 sections per mice, and 7 to 11 mice per group. *P*_G_: *P* value for the effect of the genotype; *P*_Tr_: *P* value for the effect of treatment; *P*_i_: *P* value for treatment-by-genotype interaction.

To determine the effect of treatment with WNT974 on myocardial fibrosis, CVF was calculated on myocardial sections stained with picrosirius red and compared among the groups. As shown in low and high magnification fields in Figure 3 (panels A and B, respectively), the 6 months old *Myh6-Cre:Dsp*^W/F^ mouse hearts exhibited about 2-fold increase in the CVF as compared to the corresponding *WT* mouse hearts (1.98% ± 0.45% *vs.* 1.1% ± 0.18 %, respectively, *P* = 0.0005). Whereas treatment with WNT974 had no effects on myocardial CVF in the *WT* mice, it significantly reduced the CVF in the *Myh6-Cre:Dsp*^W/F^ mice to normal values (WNT974: 1.4% ± 0.34% *vs.* untreated: 1.98% ± 0.45 %, *P* = 0.005). Genotype-by-treatment interaction analysis showed a significant interaction between the genotypes and the response to therapy, largely reflective of the genotype-dependent effects of the WNT974 on the CVF [Figure 3C].

**Figure 4.**
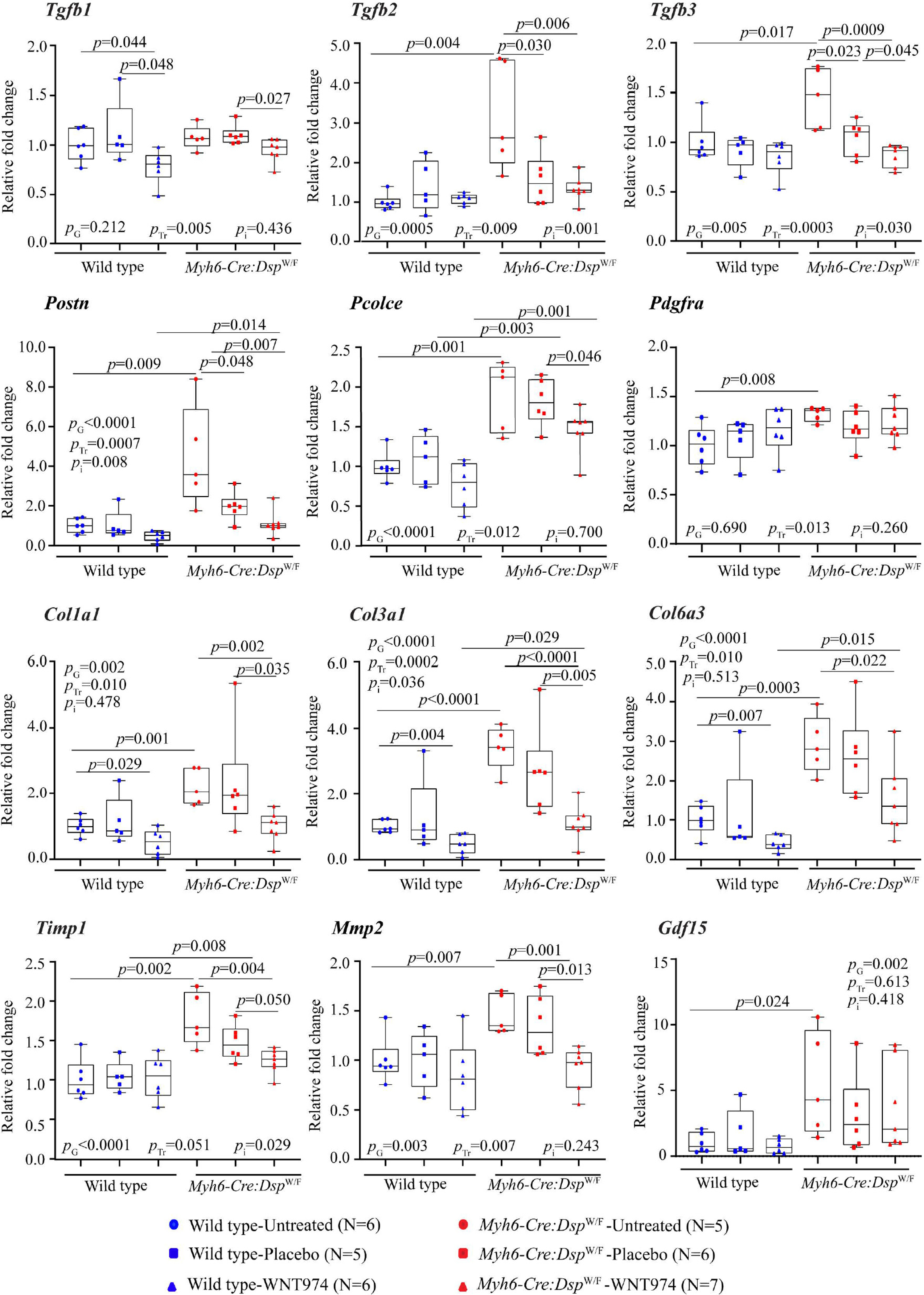
Effects of suppression of the cWNT pathway on the transcript levels of selected genes involved in myocardial fibrosis. Transcript levels of several genes involved in myocardial fibrosis were quantified by RT-PCR in the experimental groups (*N* = 5 to 7 mice per group). Treatment with WNT974 suppressed and normalized transcript levels of the majority of the pro-fibrotic genes that were upregulated in *Myh6-Cre:Dsp*^W/F^ mouse hearts. *Tgfb*: transforming growth factor β; *Postn*: periostin; *Pcolce*: procollagen C-endopeptidase enhancer; *Pdgfra*: platelet derived growth factor α; *Col1a1*, *Col3a1*, and *Col6a3*: collagen 1α1, collagen 3α, and collagen 6α3, respectively; *Timp1*: tissue inhibitor of metalloproteinase 1; *MMP2*: matrix metallopeptidase 2; *Gdf15*: growth differentiation factor 15. *P*_G_: *P* value for the effect of the genotype; *P*_Tr_: *P* value for the effect of treatment; *P*_i_: *P* value for treatment-by-genotype interaction.

To test for the corroboration of the histological findings, transcript levels of selected genes involved in cardiac fibrosis were quantified by RT-PCR and compared. Transcript levels of *Tgfb2*, *Tgfb3*, *Postn*, *Pcolce*, *Pdgfra*, *Col1a1*, *Col1a3*, *Col6a3*, *Timp1*, *Mmp2* and *Gdf15* were significantly increased in the *Myh6-Cre:Dsp*^W/F^ mouse hearts as compared to the WT controls [Figure 4]. Treatment with WNT974 reduced and even normalized elevated levels of the pro-fibrotic genes in the *Myh6-Cre:Dsp*^W/F^ mouse hearts [Figure 4]. Genotype-by-treatment interaction analysis, showed genotype-dependent salutary effects of treatment with WNT974 on CVF [Figure 4].

### Myocardial adipocytes

**Figure 5.**
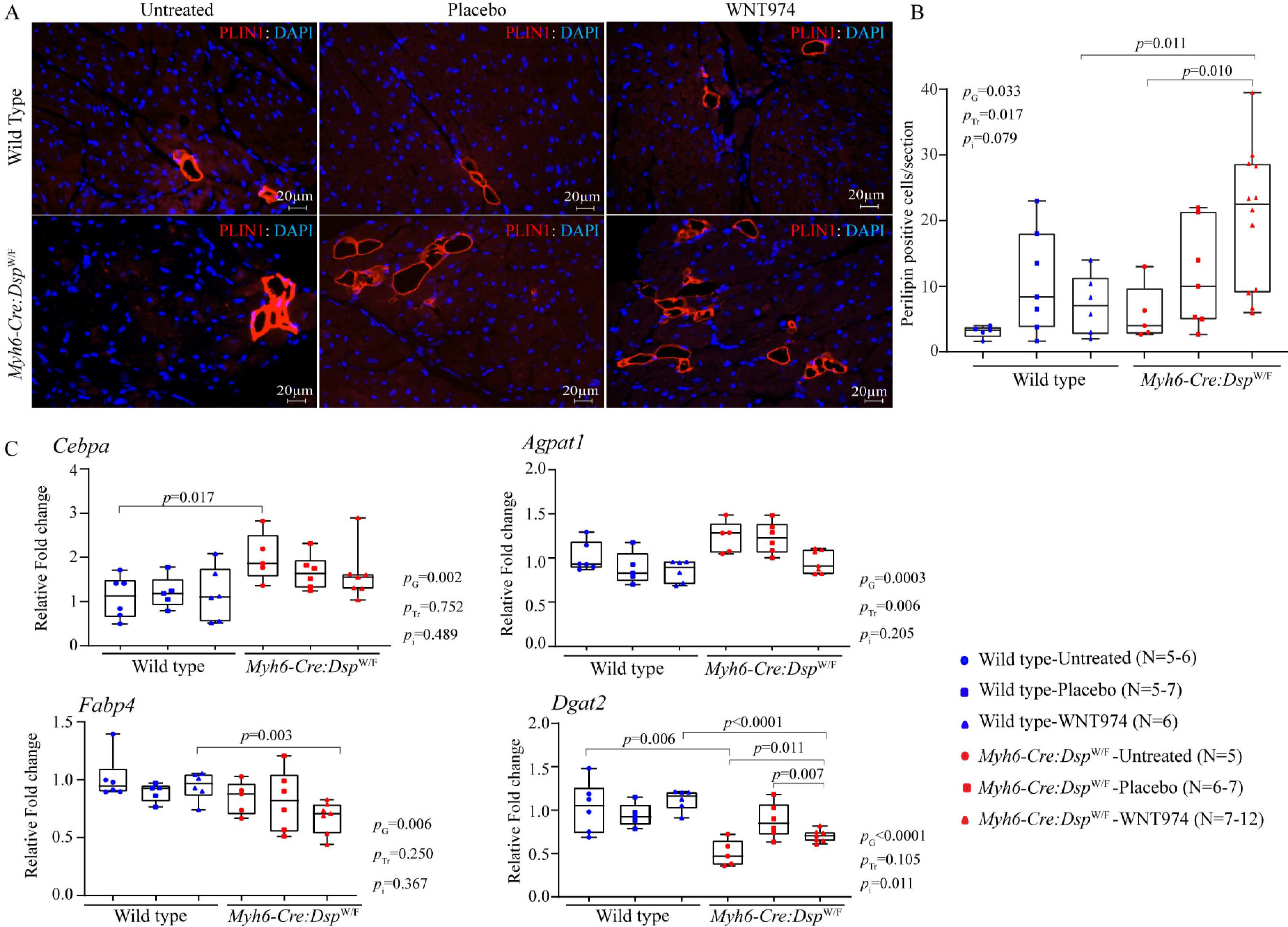
Effects of suppression of the cWNT pathway on adipogenesis. (A) Immunofluorescence panels depicting myocardial adipocytes as detected by the expression of perilipin-1 (PLIN1), a marker for mature adipocytes, in the experimental groups. (B) The mean number of adipocytes in each myocardial section was determined and compared among the experimental groups, showing an increased number of adipocytes upon inhibition of the cWNT pathway in the *Myh6-Cre:Dsp*^W/F^ mice (*N* = 5–12). (C) Transcript levels of *Cebpa* (CCAAT enhancer-binding protein α), *Agpat1* (1-acylglycerol-3-phosphate O-acyltransferase 1), *Fabp4* (fatty acid-binding protein 4), and *Dgat2* (diacylglycerol O-Acyltransferase 2) genes involved in adipogenesis, as quantified by RT-PCR in the experimental groups (*N* = 5–7). *P*_G_: *P* value for the effect of the genotype; *P*_Tr_: *P* value for the effect of treatment; *P*_i_: *P* value for treatment-by-genotype interaction.

To determine whether the suppression of the WNT signaling pathway, a known regulator of adipogenesis, affects the number of adipocytes in the *Myh6-Cre:Dsp*^W/F^ mouse heart, thin myocardial sections were stained for *PLIN1* expression^[39]^. The *Myh6-Cre:Dsp*^W/F^ mice did not show a significant increase in the number of adipocytes in the heart as compared to the WT mice [Figure 5A and B]. However, treatment with WNT974 increased the number of adipocytes in the *Myh6-Cre:Dsp*^W/F^, as compared to the WT or untreated *Myh6-Cre:Dsp*^W/F^ mice [Figure 5A and B]. In contrast, WNT974 administration had no effects on the number of adipocytes in the *WT* mouse hearts. Genotype-by-treatment interaction analysis showed significant effects of genotype and WNT974 treatment as well as a genotype-dependent effect of WNT974 [Figure 5]. To test for corroboration of the histological findings, transcript levels of selected genes involved in triglyceride synthesis were quantified by RT-PCR in the whole heart RNA extracts from the experimental groups. Transcript levels of *Cebpa* gene were increased and those of *Dgat2* were reduced in the *Myh6-Cre:Dsp*^W/F^ hearts as compared to the WT hearts, whereas transcript levels of *Agpat1* and *Fabp4* were unchanged [Figure 5C]. Treatment with WNT974 reduced transcript levels of *Agpat1* and *Fabp4* and increased *Dagt2* transcript levels, which was mainly in the *Myh6-Cre:Dsp*^W/F^ group [Figure 5C].

### Cardiac myocyte size

**Figure 6.**
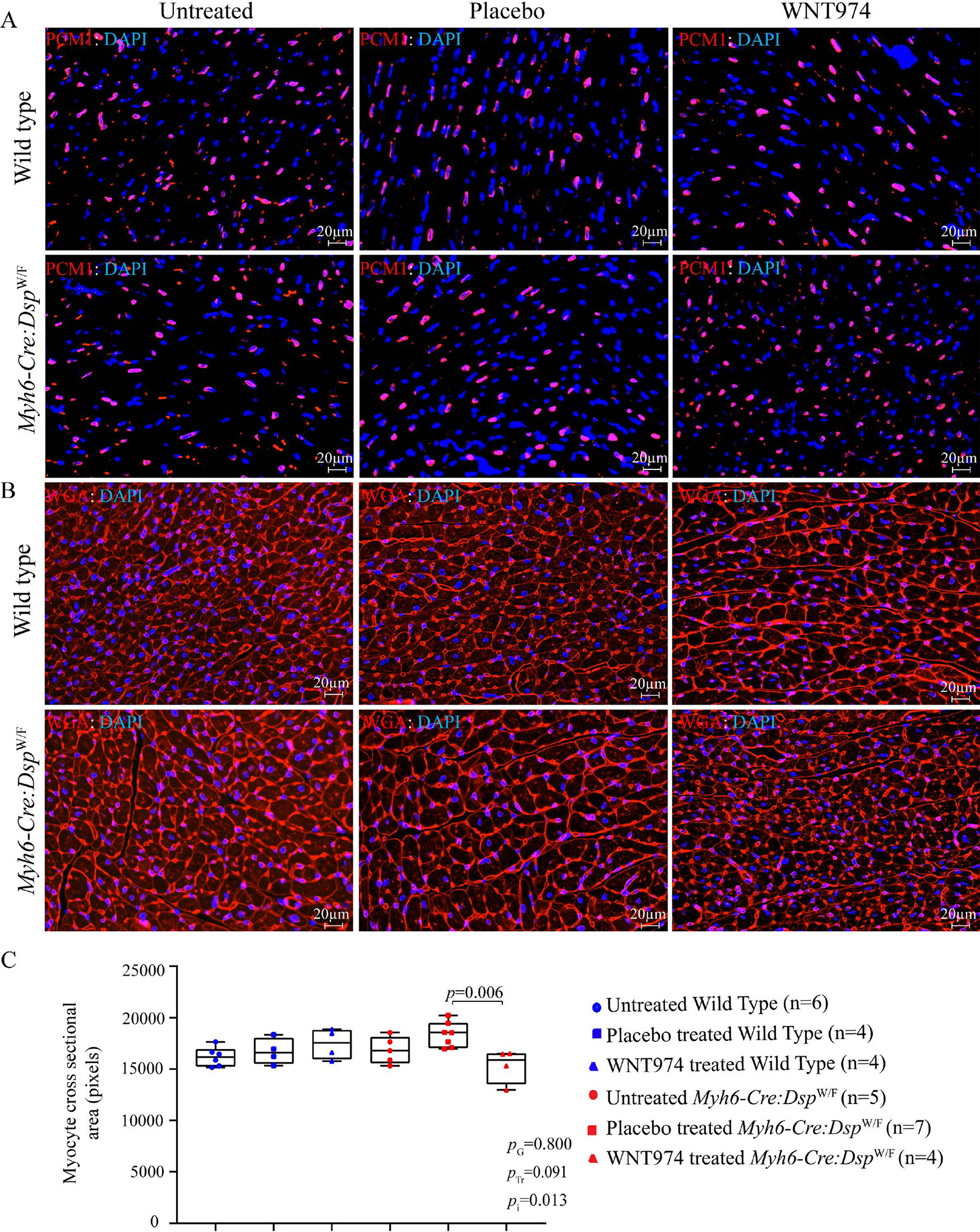
Effects of suppression of the cWNT pathway on cardiac myocyte size. (A) Representative immunofluorescence panels showing myocardial cells stained with PCM1, which marks the cardiac myocyte nuclei in the heart. (B) Thin myocardial sections stained for Wheat germ agglutinin (WGA), which stains the extra-cellular matrix and cell boundaries. (C) Quantitation of WGA-stained area presented as pixels and corrected for the number of cardiac myocytes; the latter identified by the PCM1 (pericentriolar material 1) expression in the nuclei (*N* = 4–7). *P*_G_: *P* value for the effect of the genotype; *P*_Tr_: *P* value for the effect of treatment; *P*_i_: *P* value for treatment-by-genotype interaction.

Given the potential effects of the WNT on cell size and proliferation, cardiac myocyte size was determined upon WGA staining of thin myocardial section and co-staining with PCM1, the latter to identify the myocytes. Myocyte cross-sectional area was not different between untreated *WT* and *Myh6-Cre:Dsp*^W/F^ mice [Figure 6]. However, treatment with WNT974 reduced myocyte cross-sectional area in the *Myh6-Cre:Dsp*^W/F^ mice as compared to the untreated or placebo group [Figure 6]. Genotype-by-treatment interaction analysis showed significant genotype-dependent effect of WNT974 on cardiac myocyte size in *Myh6-Cre:Dsp*^W/F^ mice [Figure 6].

### Myocardial apoptosis

**Figure 7.**
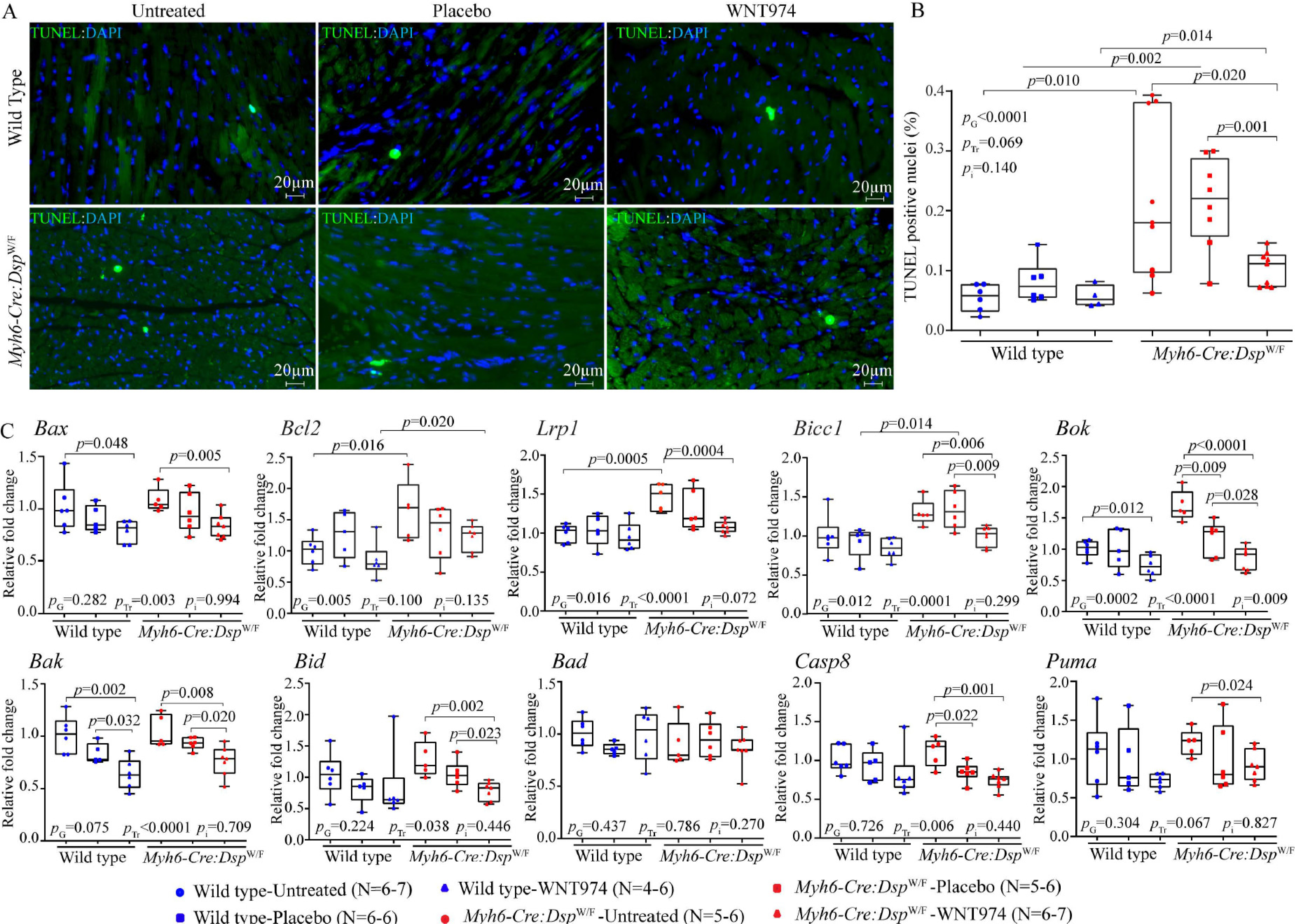
Effects of suppression of the cWNT pathway on myocardial apoptosis. (A) Thin myocardial sections showing apoptosis as detected by the TUNEL assay in the experimental groups. (B) Quantitative data on the number of cells stained positive for the TUNEL assay in the experimental groups (*N* = 6–7). (C) Quantitative RT-PCR data showing transcript levels of genes involved in apoptosis in the experimental groups (*N* = 5–7). RT-PCR: Reverse transcription-polymerase chain reaction; *Bax*: BCL2 associated X; *Bcl2*: B-cell lymphoma 2; *Lrp1*: low-desntiy lipoprotein receptor related 1; *Bicc1*: BicC family RNA binding protein 1; *Bok*: BCL2-related ovarian killer; *Bak*: BCL2-antagonist/killer 1; *Bid*: BH3 interacting domain death agonist; *Bad*: BCL2-associated agonist of cell death; *Casp8*: caspase 8; *Puma* (also known as BBC3): BCL2 binding component 3. *P*_G_: *P* value for the effect of the genotype; *P*_Tr_: *P* value for the effect of treatment; *P*_i_: *P* value for treatment-by-genotype interaction.

To determine effects of suppression of the WNT pathway on myocardial apoptosis, thin myocardial sections were stained to detect apoptotic DNA fragmentations by the TUNEL assay^[21,30]^. The number of nuclei stained positive for the TUNEL assay was increased in the untreated or placebo-treated *Myh6-Cre:Dsp*^W/F^ mice compared to the corresponding WT groups [Figure 7A and B]. Treatment with WNT974 reduced and normalized the number of TUNEL-positive nuclei in the *Myh6-Cre:Dsp*^W/F^ mice [Figure 7A and B]. Treatment-by-genotype interaction analysis showed significant effects of the genotypes and WNT974 administration on the number of apoptotic cells in the myocardium [Figure 7A and B].

To corroborate the findings, transcript levels of selected genes involved in apoptosis were quantified by RT-PCR and compared among the groups. Transcript levels of *Bcl2*, *Bicc1*, *Lrp1*, *Bax*, *Bok*, *Bak*, *Bid*, *Casp8* and *Puma* were upregulated in the hearts of *Myh6-Cre:Dsp*^W/F^ mouse hearts as compared to the WT mouse hearts [Figure 7C]. Treatment with WNT974 reduced transcript levels of several genes involved in apoptosis in the *Myh6-Cre:Dsp*^W/F^ as well as the WT mouse hearts, albeit more prominently and consistently in the *Myh6-Cre:Dsp*^W/F^ mice [Figure 6C]. Treatment-by-genotype interaction analysis identified the predominant effect of the genotypes, the WNT974 administration, and to a lesser extent genotype-dependent effects of treatment with WNT974 on the transcript levels of genes involved in cell apoptosis [Figure 6C].

## DISCUSSION

The cWNT pathway has been implicated in the pathogenesis of ACM, particularly in fibro-adipogenesis, which is the histological hallmark of ACM, for over a decade^[21]^. However, whether the observed changes are pathogenic or compensatory has remained unsettled. Data in the present study suggest that suppression of the WNT imparts partially beneficial effects. Specifically, administration of WNT974, an established inhibitor of the WNT pathway, improves cardiac function, attenuates myocardial fibrosis, and reduced apoptosis. In contrast, suppression of the WNT pathway was associated with an increased number of adipocytes in the ventricular myocardium. Suppression of the WNT had no significant effect on cardiac arrhythmias or the survival rates of the *WT* or *Myh6-Cre:Dsp*^W/F^ mice, albeit the study was not designed to detect differences in the survival rates. The findings suggest interventions targeting to suppress the WNT in ACM might impart beneficial effects on myocardial phenotype in ACM.

The phenotypic consequences of the suppression of the WNT in the *Myh6-Cre:Dsp*^W/F^ mice were concordantly observed by complementary techniques and across multiple phenotypes. Likewise, the findings of the study on reduced myocardial fibrosis and apoptosis and increased myocardial adipocytes in an ACM model are in accord with the known biological effects of the WNT signaling pathway^[26,39,40]^. Activation of the WNT is known to prevent adipogenesis and enhance fibrosis, conversely, its suppression is known to provoke adipogenesis and attenuate fibrosis^[26,39,40]^. Likewise, data in the present study showing improvement in cardiac function and reduced apoptosis in the *Myh6-Cre:Dsp*^W/F^ mice upon treatment with WNT974 are in accord with the role of the WNT in regulating apoptosis, cardiac remodeling, and cardiac function post-ischemic injury (reviewed in^[41]^). Overall, the findings of the present study, validated by complementary methods, are in accord with the biological functions of the cWNT pathway and its role as a major transcriptional switch between adipogenesis and fibrogenesis as well as a regulator of cell death and cardiac function (reviewed in^[41]^).

The intervention was started prior to the onset of cardiac dysfunction or fibrosis in the 3-month-old mice, and the WNT974 was administered daily for another 3 months, assuming that at 6 months of age, the *Myh6-Cre:Dsp*^W/F^ mice in the untreated or placebo groups would show a phenotype, based on the previous observations^[21,30]^. Therefore, the study by design might be considered a preventive study rather than a reversal of the phenotype (treatment) study. As expected, and in accord with the published data, the 6-month-old *Myh6-Cre:Dsp*^W/F^ mice exhibited an ACM-like phenotype, albeit the observed phenotype was not severe and survival was normal. Administration of WNT974 was effective in preventing and attenuating the age-dependent evolution of the phenotype. A longer duration of the intervention would be necessary to detect potential effects or the lack thereof the suppression of the WNT pathway on survival in the *Myh6-Cre:Dsp*^W/F^ mice. Likewise, a study in older mice with fully blown ACM-like phenotype would enable detecting potential effects of the suppression of the WNT pathway on reversal of the established phenotype. Finally, WNT974 inhibits porcupine, which is responsible for palmitoylation and subsequent transfer and secretion of the WNT molecule^[32]^. Therefore, it suppresses both the cWNT and the non-canonical pathways. Therefore, whether the observed beneficial effects are the consequences of the suppression of the cWNT or the non-canonical WNT pathway cannot be discerned.

Whereas the findings of the present study indicate beneficial levels of the suppression of the WNT pathway in a mouse model of ACM, others have reported beneficial effects of activation of inhibition of glycogen synthase kinase 3 (GSK3β), which is expected to lead to activation of the cWNT pathway, in ACM models^[28,29,42,43]^. The interventions are considerably different, as the present study is the first to use WNT974, which specifically and effectively suppresses the WNT pathway, whereas the previous studies targeted GSK3β, which has numerous functions, in addition to targeting the β-catenin of the cWNT pathway for phosphorylation and degradation by ubiquitination (reviewed in^[44]^). Genetic mouse models whereby the cWNT pathway is either activated or suppressed in the ACM models would be expected to provide more compelling evidence on the effects of targeting the cWNT pathway in ACM.

The study has a number of limitations, including a relatively modest sample size of the mice in the survival analysis. There was a modest decrease in the survival rate of mice treated with WNT974, which was not statistically significant. Likewise, several indices of cardiac size and function showed significant improvement, whereas the differences among other indices did not reach statistical significance, but they trended in the same direction (toward improvement). In addition, cardiac rhythm was monitored only briefly, which might be insufficient to detect uncommon arrhythmias. Thus, the lack of a statistically significant phenotypic effect on certain indices, such as the survival rates, might be subject to type II statistical errors, requiring a much larger study or a longer duration of the intervention.

In conclusion, the data suggest potential beneficial effects of the suppression of the cWNT signaling pathway on prevention and attenuation of cardiac phenotype in a mouse model of ACM caused by *Dsp-*haploinsufficiency. The findings set the stage for genetic intervention and large studies to confirm the potential beneficial effects of the suppression of the cWNT in ACM.

## Supplementary Material

Supplementary Material

## Figures and Tables

**Table 1.** Echocardiographic phenotype at the completion of the study

	WT			*Myh6-Cre:Dsp* ^W/F^			2-way ANOVA		

	Untreated	DMSO	WNT974	Untreated	DMSO	WNT974	*P* Genotype	*P* Treatment	*P* Interaction
N	10	9	10	10	10	14	NA	NA	NA
M/F	5/5	4/5	6/4	4/6	5/5	7/7	NA	NA	NA
Age (days)	189.30 ± 10.44	185.33 ± 7.50	184.70 ± 5.54	191.60 ± 18.40	187.00 ± 3.37	187.93 ± 8.12	0.306	0.336	0.985
Body weight (g)	32.02 ± 4.67	31.03 ± 4.34	29.15 ± 4.82	34.87 ± 6.49	35.09 ± 7.99	32.27 ± 4.11	0.020	0.214	0.938
HR (bpm)	501.15 ± 55.08	530.24 ± 41.48	491.38 ± 45.67	513.81 ± 52.57	514.41 ± 35.28	520.18 ± 28.86	0.441	0.419	0.254
LVAWT (mm)	0.52 ± 0.05	0.51 ± 0.05	0.48 ± 0.04	0.55 ± 0.07	0.55 ± 0.08	0.51 ± 0.04	0.039	0.035	0.871
LVPWT (mm)	0.53 ± 0.04	0.51 ± 0.04	0.48 ± 0.04	0.55 ± 0.08	0.55 ± 0.08	0.50 ± 0.04	0.055	0.021	0.852
LVEDD (mm)	3.43 ± 0.27	3.47 ± 0.35	3.43 ± 0.44	3.55 ± 0.51	3.86 ± 0.26	3.62 ± 0.15	0.009	0.246	0.485
LVEDDI (mm/g)	0.11 ± 0.02	0.11 ± 0.01	0.12 ± 0.02	0.11 ± 0.03	0.11 ± 0.03	0.11 ± 0.02	0.629	0.266	0.825
LVESD (mm)	2.00 ± 0.17	2.05 ± 0.31	2.05 ± 0.36	2.43 ± 0.63	2.57 ± 0.43	2.15 ± 0.23	0.0006	0.197	0.164
LVFS (%)	41.65 ± 1.77	41.10 ± 4.69	40.56 ± 4.75	32.38 ± 8.63	33.74 ± 7.48	40.68 ± 5.53	0.0005	0.094	0.026
LVM (mg)	41.35 ± 6.66	41.81 ± 10.73	38.11 ± 11.38	46.99 ± 12.21	54.43 ± 11.72	43.47 ± 5.55	0.003	0.061	0.422
LVMI (mg/g)	1.30 ± 0.16	1.33 ± 0.21	1.29 ± 0.25	1.36 ± 0.34	1.56 ± 0.15	1.35 ± 0.13	0.036	0.138	0.388

WT: Wild type; DMSO: dimethyl sulfoxide; M/F: male/female; HR: heart rate; LVAWT: left ventricular anterior wall thickness; LVPWT: left ventricular posterior wall thickness; LVEDD: left ventricular end diastolic diameter; LVEDDI: left ventricular end diastolic diameter indexed to body weight; LVESD: left ventricular end systolic diameter; LVFS: left ventricular fractional shortening; LVM: left ventricular mass; LVMI: left ventricular mass indexed to body weight.

## Data Availability

Not applicable.
