# Supplementary material for: Pharmacological suppression of the WNT signaling pathway attenuates age-dependent expression of the phenotype in a mouse model of arrhythmogenic cardiomyopathy
Source: J Cardiovasc Aging. Author manuscript; Available in PMC 2021 Aug 25. (PMC8386676; doi:10.20517/jca.2021.04)
Supplement: Supplementary Material [file NIHMS1713023-supplement-Supplementary_Material.pdf]

Online Supplementary Material

Pharmacological Suppression of the WNT Signaling Pathway Attenuates Age-Dependent Expression of the Phenotype in a Mouse Model of Arrhythmogenic Cardiomyopathy

Sirisha M. Cheedipudi*, Siyang Fan*, Leila Rouhi, Ali J. Marian§

Center for Cardiovascular Genetics, Institute of Molecular Medicine and Department of Medicine, University of Texas Health Sciences Center at Houston, Texas 77030.

Short title: Beneficial effects of suppression of the WNT in ACM

*Co first authors

§ Address for Correspondence:

AJ Marian, M.D.

Center for Cardiovascular Genetics

6770 Bertner Street

Suite C900A

Houston, TX 77030

713 500 2350

ORCiD: 0000-0002-1252-7120

Ali.J.Marian@uth.tmc.edu

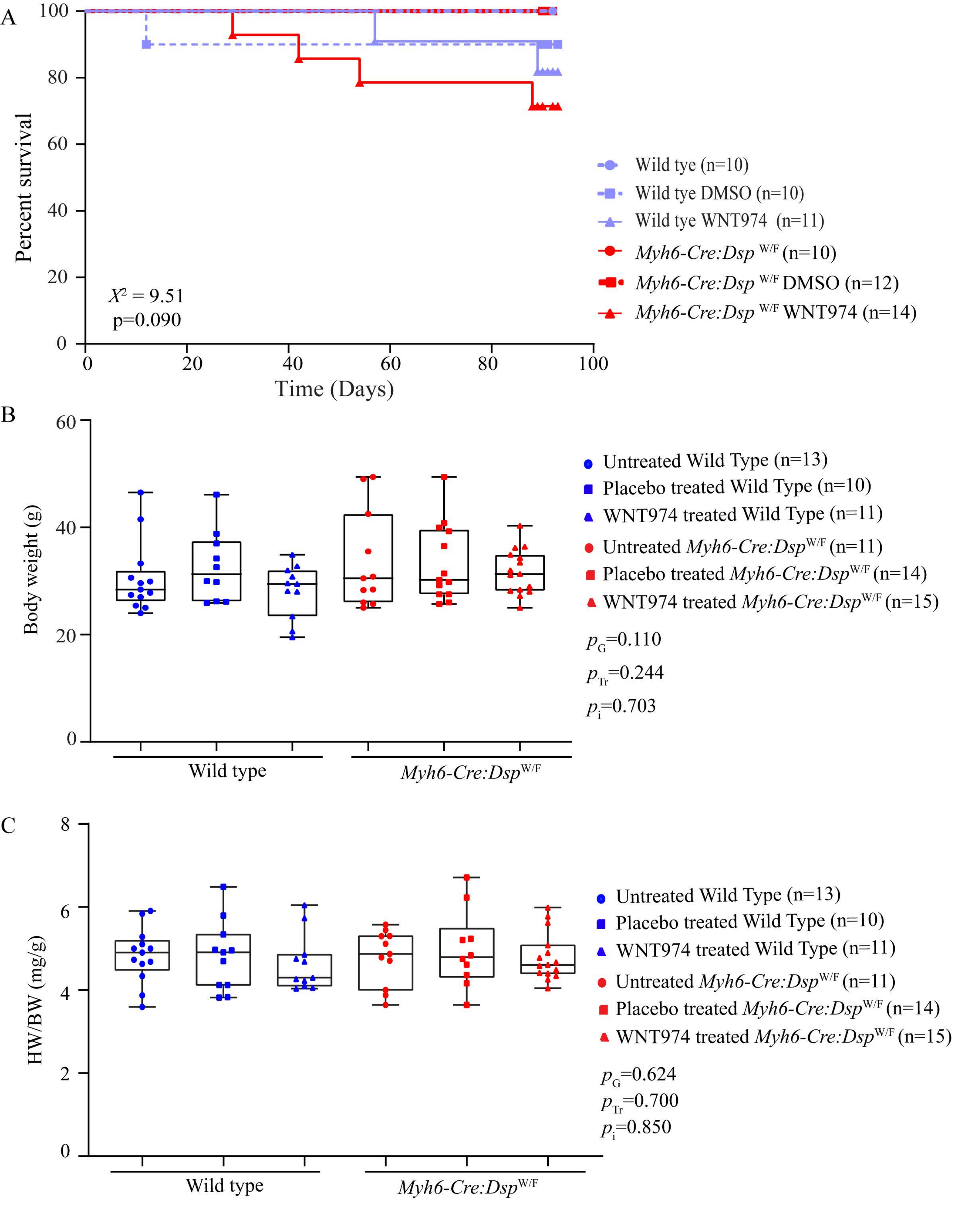

Online Figure 1: Survival and gross morphology of untreated, placebo and WNT974 treated wild type and *Myh6-Cre:Dsp*$^{W/F}$ mice.

A. Kaplan Meier survival plots; **B.** Body weight, and **C.** Heart weight to body weight ratio in untreated, placebo treated, and WNT974 treated wild type and*Myh6-Cre:Dsp*$^{W/F}$ mice.

Online Table I

A. Primer sequences used for genotyping:

Transgene	Sequence
Myh6-Cre	Forward: ATGACAGACAGATCCCTCCTATCTCC<br>Reverse: GCGAACCTCATCACTCGTTGCATCGA
Dsp$^{F/F}$	Forward: TAAGCTCCCCTCACTTCTCCAGTC<br>Reverse: TTCTCTTTGTCTGTTGCCATGTGA

B. Primer sequences used in qPCR reactions

Name	Sequence
Gapdh	Forward: AACTTTGGCATTGTGGAAGG<br>Reverse: GGATGCAGGGATGATGTTCT
Lrp1	Forward: CAAAGCTGAAGGCTCCGAGT<br>Reverse: TATGCGGACACTCTCATCGC
Tgfb1	Forward: TGGAGCAACATGTGGAACTC<br>Reverse: GTCAGCAGCCGGTTACCA
Tgfb2	Forward: AGGAGTGGCTTCACCACAAAGACA<br>Reverse: ATTAGACGGCACGAAGGTACAGCA
Tgfb3	Forward: AGCTCTTCCAGATACTTCGACC<br>Reverse: AAAGACAGCCATTCAGCGGT
Col6a3	Forward: GATCGCTTTCGACTCCTCCC<br>Reverse: TGTTTACGTGAACTTCCGTGGT
Timp1	Forward: CATGGAAAGCCTCTGTGGATA<br>Reverse: CTCAGAGTACGCCAGGGAAC
Postn	Forward: AGAGAAATCCCTGCACGACA<br>Reverse: GTTGGTGCAAACAAGGTCCA
Gdf15	Forward: AACCCCTGGTCTGGGGATAC<br>Reverse: CATGTCGCTTGTGTCCTTTCAG
Pcolce	Forward: GGGCACTGAGCACCAGTTTT<br>Reverse: GCTGATGCCTGGTGGGTAAT
Pdgfra	Forward: GGAACCTCAGAGAGAATCGGC<br>Reverse: CATAGCTCCTGAGACCCGCT
Bcl2	Forward: CACCCCTGGTGGACAACATC<br>Reverse: GTTCCACAAAGGCATCCCAGC
Bicc1	Forward: GCTGGCAATCTCAGAGCTAA<br>Reverse: TGACGCTCGCAATGTCTGAA
Casp8	Forward: GCGTGGAACAGGAAGTGAGTA<br>Reverse: GAAGAGCTGTAACCTGTGGC
Bok1	Forward: CAGCGTATACCGGAACGTGG<br>Reverse: TTGCCCCATGTGATACCTGC
Bak1	Forward: CAAGATCGCCTCCAGCCTATT

	Reverse: CCCAGGAAGCCGGTCAAAC
Bid1	Forward: CCGCAAACCTTTGCCTTAGC<br>Reverse: AACCGTTGCTGACCTCAGAGT
Bad1	Forward: AAATGGGAACCCCAAAGCAG<br>Reverse: GAACATACTCTGGGCTGCTGG
Agpat1	Forward: GACAGAGATACAGCCAGCCG<br>Reverse: GCTCCATTCTGGTCACCTCA
Puma	Forward: GAGACAAGAAGAGCAGCATCG<br>Reverse: TAGTTGGGCTCCATTTCTGG
Bax	Forward: ACAGGGGCCTTTTTGCTACA<br>Reverse: CACTCGCTCAGCTTCTTGGT

C. TaqMan assays

Gene	TaqMan Assay ID
Gapdh	Mm99999915_g1
Col1a1	Mm00801666_g1
Col3a1	Mm00802300_m1
Mmp2	Mm00439498_m1
Axin2	Mm00443610_m1
Myh6	Mm00440359_m1
Nppa	Mm01255747_g1
Nppb	Mm01255770_g1
Acta1	Mm00808218_g1
Cebpa	Mm00514283_s1
Fabp4	Mm00445878_m1
Dgat2	Mm00499536_m1

D. Antibodies used in the study

Antibodies	Concentration	Supplier	Catalogue number
Goat anti-Rabbit IgG, Alexa Fluor 594	1:1000 (IF)	Invitrogen	A21208
DAPI	1:1000 (IF)	Sigma	D8417
PLIN1	1:100 (IF)	Cell Signaling Technology	9349
TCF7L2	1:200(IF)	Cell Signaling Technology	2569
PCM1	1:500(IF)	Sigma	HPA023370
WGA (594 conjugated)	1μg/ml (IF)	Thermo Scientific	W21405

Online Table 2

Echocardiographic Data at the Onset of the Intervention (3 months old mice)

	WT			*Myh6-Cre:Dsp*$^{W/F}$			2-way ANOVA		
	Untreated	DMSO	WNT-974	Untreated	DMSO	WNT-974	P Genotype	P Treatment	P Interaction
N	14	9	10	13	10	14	NA	NA	NA
M/F	7/7	4/5	6/4	6/7	5/5	7/7	NA	NA	NA
Age (days)	99.93±5.15	97.22±7.28	96.40±4.55	98.54±6.42	97.70±3.62	97.64±7.32	0.940	0.384	0.723
Body weight (g)	29.39±4.34	27.36±5.42	27.96±3.51	28.91±4.33	28.55±6.02	30.76±4.29	0.298	0.582	0.456
HR (bpm)	506.47±35.60	492.90±31.96	495.60±32.32	499.05±32.52	502.48±42.67	499.85±38.98	0.808	0.851	0.711
LVAWT (mm)	0.62±0.05	0.59±0.05	0.63±0.13	0.60±0.03	0.56±0.05	0.56±0.07	0.039	0.340	0.376
LVPWT (mm)	0.64±0.03	0.61±0.11	0.70±0.16	0.61±0.03	0.62±0.10	0.63±0.11	0.202	0.233	0.386
LVEDD (mm)	3.39±0.23	3.43±0.39	3.37±0.53	3.65±0.46	3.52±0.46	3.55±0.30	0.0702	0.856	0.781
LVEDDI (mm/g)	0.12±0.02	0.13±0.02	0.12±0.01	0.13±0.02	0.12±0.02	0.12±0.02	0.627	0.576	0.360
LVESD (mm)	2.03±0.29	2.19±0.42	2.05±0.59	2.31±0.44	2.24±0.50	2.24±0.32	0.100	0.849	0.656
FS (%)	40.37±6.48	36.55±6.37	40.00±10.07	36.84±7.37	36.93±7.24	37.26±5.95	0.269	0.633	0.655
LV Mass (mg)	51.16±6.13	49.70±12.23	54.40±11.90	55.29±12.37	50.76±11.10	51.86±10.54	0.735	0.595	0.549
LVMI (mg/g)	1.77±0.28	1.84±0.41	1.96±0.44	1.93±0.37	1.80±0.32	1.71±0.41	0.621	0.968	0.150

Abbreviations: WT: wild type; DMSO: dimethyl sulfoxide; M/F: male/female; HR: heart rate; LVAWT: left ventricular anterior wall thickness; LVPWT: left ventricular posterior wall thickness; LVEDD: Left ventricular end diastolic diameter; LVEDDI: Left ventricular end diastolic

diameter indexed to body weight; LVESD: Left ventricular end systolic diameter; LVFS: Left ventricular fractional shortening; LVM: left ventricular mass; LVMI: Left ventricular mass indexed to body weight.
